# Polymer-based dental filling materials placed during pregnancy and risk to the foetus

**DOI:** 10.1186/s12903-018-0608-1

**Published:** 2018-08-22

**Authors:** Trine Lise Lundekvam Berge, Gunvor Bentung Lygre, Stein Atle Lie, Lars Björkman

**Affiliations:** 1 Dental Biomaterials Adverse Reaction Unit, Uni Research Health, Bergen, Norway; 2 Oral Health Centre of Expertise in Western Norway, Bergen, Hordaland Norway; 3 Department of Clinical Dentistry, University of Bergen, Bergen, Norway

**Keywords:** Polymer-based dental filling materials, Pregnancy, Adverse birth outcomes, Congenital malformation, Birth weight, Stillbirth, Premature birth, Bisphenol A, BPA, The Norwegian mother and child cohort study

## Abstract

**Background:**

Tooth-coloured polymer-based dental filling materials are currently the first choice for dental restorative treatment in many countries. However, there are some concerns about their safety. It has been shown that substances known as endocrine disrupters, which might pass through the placental barrier, are released from these materials within the first hours after curing. Thus, the placement of polymer-based dental fillings in pregnant women may put the vulnerable foetus at risk. Large epidemiological studies exploring the risk of having polymer-based dental materials placed during pregnancy are lacking. The aim of this study was to investigate the association between the placement of polymer-based dental fillings during pregnancy and adverse birth outcomes.

**Methods:**

This study is based on data from the large Norwegian Mother and Child Cohort Study (MoBa). The information about dental treatment during pregnancy was obtained from questionnaires sent to the participating women during weeks 17 and 30 of pregnancy. Reported placement of “white fillings” was used as exposure marker for having received polymer-based dental filling materials. Only singleton births were included in the present study. Data were linked to the Medical Birth Registry of Norway. Logistic regression models that included the mother’s age, level of education, body mass index, parity, and smoking and alcohol consumption during pregnancy were used to estimate the odds ratio (OR) and 95% confidence interval (CI). Different adverse birth outcomes were of interest in the present study.

**Results:**

Valid data were available from 90,886 pregnancies. Dentist consultation during pregnancy was reported by 33,727 women, 10,972 of whom had white fillings placed. The adjusted logistic regression models showed no statistically significant association between having white dental fillings placed during pregnancy and stillbirth, malformations, preterm births, and low or high birth weight.

**Conclusions:**

In this study, women who reported white fillings placed during pregnancy had no increased risk for adverse birth outcomes compared with women who did not consult a dentist during pregnancy. Thus, our findings do not support the hypothesis of an association between placement of polymer-based fillings during pregnancy and adverse birth outcomes.

## Background

Tooth-coloured polymer-based materials are the first choice for dental restorative treatment in many countries [1, 2]. However, there are concerns about the safety of these materials [3]. Results of in vitro and in vivo studies have shown that substances that potentially could lead to adverse effects in the patient are released from these materials within 24 h after curing [4–8]. Elution may initially be due to incomplete polymerization [9, 10] and contaminants [11, 12]. The local adverse effects [13] caused by the leachable components are rare [14]. However, the possibility of systemic adverse effects could not be ruled out [15].

The elution of bisphenol A (BPA) has been of particular concern [16]. BPA is a chemical known to be an endocrine disruptor, mimicking oestrogen [17, 18]. Polymer-based dental filling materials may contain BPA as an impurity from the production process of bisphenol-A glycidyl dimethacrylate (Bis-GMA) [8, 11, 19, 20] or, less probable, a degradation product of monomers [12, 21, 22]. Results from animal studies have indicated that BPA has reproductive, developmental and systemic toxic effects [23, 24]. It has been shown that newly placed composite restorations in humans may be associated with short-term elevated BPA levels in both saliva and urine [4, 7].

The impact of exposure to BPA on human health remains uncertain. However, data from the literature indicate that exposure to BPA, even at relatively low doses, could potentially result in adverse health effects [15]. Moreover, studies suggest that BPA might pass through the placental barrier [25], and thus, maternal exposure to BPA may offer a potential risk to the vulnerable foetus.

Even though substances with potential toxicity are released from dental polymer-based materials [4, 5], studies exploring the risk of having these materials placed during pregnancy are lacking.

The aim of the present study was to investigate whether the placement of polymer-based dental fillings during pregnancy is associated with adverse birth outcomes including stillbirth, preterm birth, malformations and low or high birth weight.

## Methods

Data from the ongoing Norwegian Mother and Child Cohort Study (MoBa), a prospective population-based cohort study conducted by the Norwegian Institute of Public Health, were used. From 1999 to the end of 2008, pregnant women in Norway were invited to MoBa through a postal invitation in connection with their first routine ultrasound examination. The participation rate was approximately 41%, and the cohort currently comprises more than 108,000 pregnancies, 114,000 children, 95,000 mothers and 75,000 fathers. Written informed consent was obtained from each participant upon recruitment [26, 27].

In the present study, data were gathered from two questionnaires that were sent to the participating women in weeks 17 and 30 of pregnancy [28]. Each woman could participate with multiple pregnancies. Only singleton births were included in the present study.

Information about white fillings placed during pregnancy was obtained from the questionnaires sent to the participants in week 30. Reported placement of white fillings was used as exposure marker. The participants reported if they had consulted a dentist during pregnancy (“Have you been to the dentist during this pregnancy? Yes/No”) and if so, whether they had received white fillings (“If, yes, did the dentist put in new white fillings? Yes/No”).

**Fig. 1:**
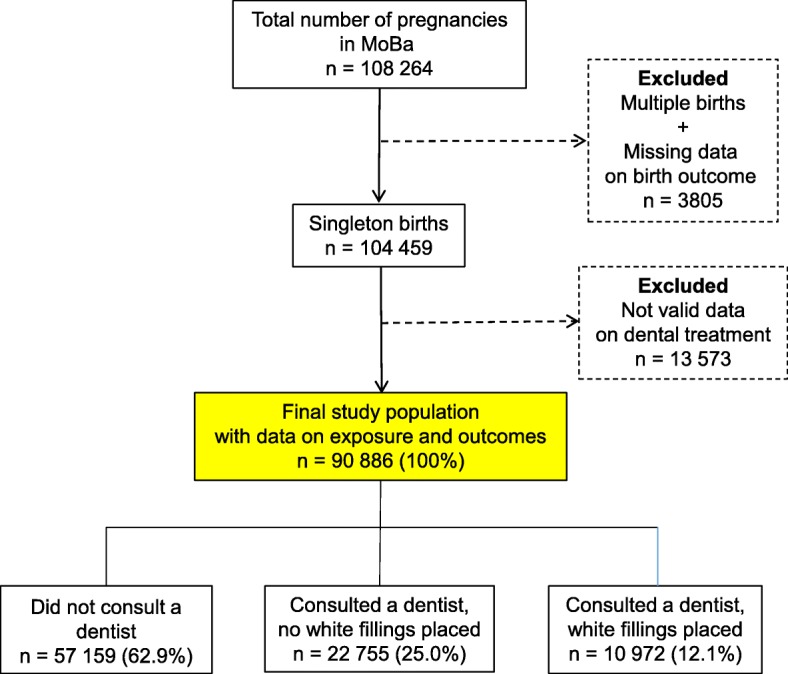
Flowchart showing number of participants included in the study and the groups available for analysis

Women without valid information about dental treatment during pregnancy and those with missing data on birth outcomes were excluded, leaving a study population that included 90,886 pregnancies (Fig. 1).

Information about gender, preterm delivery, stillbirth, malformations, birth weight and mother’s age at delivery was obtained from the Medical Birth Registry of Norway (MBRN) [29]. The mother’s 11-digit unique personal identification number assigned to every citizen in Norway was used to link data sources. Gestational age was based on ultrasound examination in the 17th week of pregnancy.

Infants were classified as late preterm if they were born between gestational week 33 and 37, and very preterm if they were born before or during the 32nd gestational week [30, 31]. Infants with a birth weight less than 2500 g at birth were classified as low-birth weight infants, and infants with a birth weight more than 4000 g were classified as high-birth weight infants [32].

Maternal body mass index (BMI; kg/m^2^) was calculated from self-reported pre-pregnancy height and weight. The BMI was categorized according to the WHO classification [33].

Information about parity, defined as the number of former births with a gestational age of 12 weeks or more, was based on data reported by the mothers in the MoBa study and from the MBRN.

Information about education, smoking habits and alcohol consumption during pregnancy was obtained from the first questionnaire completed at approximately the 17th week.

The present study is based on version 8 of the quality-assured MoBa data files. We defined dental treatment during pregnancy as follows: participants who did not consult a dentist during pregnancy (reference category); participants who consulted a dentist but had no white fillings placed; and participants who consulted a dentist and had white fillings placed (Fig. 1).

Infants were defined as small for gestational age (SGA) if the weight at birth was less than the 10th percentile for gestational age and large for gestational age (LGA) if they were larger than the 90th percentile. Very small for gestational age was defined as weight below the 2.5th percentile [34].

The odds ratio (OR) with a 95% confidence interval was calculated using logistic regression. The OR was adjusted for maternal age (≤19, 20–24, 25–29, 30–34, 35–39, 40+ years), length of education (≤12, 13–16, ≥17 years), pre-pregnancy BMI (< 18.5, 18.5–24.9, 25.0–29.9, 30.0–34.9, 35.0–39.9, ≥ 40 kg/m^2^), parity (first, second and more), smoking during pregnancy (never, occasionally, daily) and alcohol consumption during pregnancy (never, less than once a week, once a week, more than once a week).

Analyses were performed using IBM-SPSS (IBM Corp. Released 2016. IBM SPSS Statistics for Windows, Version 24.0, Armonk, NY, USA: IBM Corp.). *p*-values less than 0.05 were considered statistically significant.

The MoBa cohort study obtained a license from the Norwegian Data Inspectorate, and this research project was approved by the Regional Ethics Committee for Medical Research (REC South-East D, 2011/727).

## Results

Dentist consultation during pregnancy was reported by 33,727 women, and of these, 10,972 had white fillings placed (Fig. 1). Detailed descriptive information regarding the characteristics of the participants is included in Table 1. Of the included pregnancies, 204 (0.2%) resulted in a stillbirth. The overall proportion of malformation was 4.8%, and the proportion of very preterm births and late preterm births was 0.6 and 3.8%, respectively (Table 2).

**Table 1:** Characteristics of the participants related to dental treatment during pregnancy (*n* = 90,886)

	Did not consult a dentist	Consulted a dentist, no white fillings placed	Consulted a dentist, white filling placed	Total
Number of participating pregnancies, *n* (%)	57,159 (62.9)	22,755 (25.0)	10,972 (12.1)	90,886 (100)
Maternal age (years), *n* (%)				
≤ 19	525 (0.9)	207 (0.9)	127 (1.2)	859 (0.9)
20–24	6056 (10.6)	1767 (7.8)	1195 (10.9)	9018 (9.9)
25–29	19,288 (33.7)	7159 (31.5)	3686 (33.6)	30,133 (33.2)
30–34	21,928 (38.4)	9273 (40.8)	3990 (36.4)	35,191 (38.7)
35–39	8315 (14.5)	3856 (16.9)	1711 (15.6)	13,882 (15.3)
≥ 40	1047 (1.8)	493 (2.2)	263 (2.4)	1803 (2.0)
Maternal pre-pregnant Body Mass Index, *n* (%)				
< 18.5	1663 (2.9)	660 (2.9)	319 (2.9)	2642 (2.9)
18.5–24.9	36,021 (63.0)	14,956 (65.7)	6655 (60.7)	57,632 (63.4)
25.0–29.9	12,003 (21.0)	4542 (20.0)	2443 (22.3)	18,988 (20.9)
30.0–34.9	3837 (6.7)	1402 (6.2)	842 (7.7)	6081 (6.7)
35.0–39.9	1091 (1.9)	354 (1.6)	232 (2.1)	1677 (1.8)
≥ 40	341 (0.6)	124 (0.5)	68 (0.6)	533 (0.6)
Missing	2203 (3.9)	717 (3.2)	413 (3.8)	3333 (3.7)
Parity, *n* (%)				
Para 0 (first pregnancy)	25,428 (44.5)	10,897 (47.9)	4836 (44.1)	41,161 (45.3)
Para 1+ (second pregnancy or more)	31,731 (55.5)	11,858 (52.1)	6136 (55.9)	49,725 (54.7)
Maternal education, *n* (%)				
≤ 12 years	18,849 (33.0)	6831 (30.0)	4177 (38.1)	29,857 (32.9)
13–16 years	22,042 (38.6)	9226 (40.5)	4018 (36.6)	35,286 (38.8)
≥17 years	12,725 (22.3)	5366 (23.6)	2097 (19.1)	20,188 (22.2)
Missing	3543 (6.2)	1332 (5.9)	680 (6.2)	5555 (6.1)
Smoking during pregnancy, *n* (%)				
Never	45,831 (80.2)	18,208 (80.0)	8420 (76.7)	72,459 (79.7)
Occasionally	1421 (2.5)	567 (2.5)	374 (3.4)	2362 (2.6)
Daily	2848 (5.0)	968 (4.3)	821 (7.5)	4637 (5.1)
Missing	7059 (12.3)	3012 (13.2)	1357 (12.4)	11,428 (12.6)
Alcohol during pregnancy, *n* (%)				
Never	42,203 (73.8)	16,731 (73.5)	7834 (71.4)	66,768 (73.5)
Less than once a week	5709 (10.0)	2512 (11.0)	1251 (11.4)	9472 (10.4)
Once a week	233 (0.4)	100 (0.4)	46 (0.4)	379 (0.4)
More than once a week	39 (0.1)	25 (0.1)	6 (0.1)	70 (0.1)
Missing	8975 (15.7)	3387 (14.9)	1835 (16.7)	14,197 (15.6)

Body Mass Index = weight (kg)/height^2^ (m)^2^

**Table 2:** Birth outcomes by dental treatment during pregnancy (*n* = 90,886)

	Did not consult a dentist	Consulted a dentist, no white fillings placed	Consulted a dentist, white filling placed	Total
Number of boys, *n* (%)	29,387 (51.4)	11,607 (51.0)	5582 (50.9)	46,576 (51.2)
Number of preterm births, *n* (%)				
Very preterm births (≤ 32 weeks)	337 (0.6)	124 (0.5)	65 (0.6)	526 (0.6)
Late preterm births (33–36 weeks)	2150 (3.8)	884 (3.9)	454 (4.1)	3488 (3.8)
Mean birth weight (g)				
Mean birth weight (SD)	3611 (546)	3603 (538)	3607 (549)	3608 (544)
Number of children with low birth weight, *n* (%)				
Low birth weight (< 2500 g)	1465 (2.6)	576 (2.5)	290 (2.6)	2331 (2.6)
Small for gestational age (SGA) 10 percentile	3660 (6.4)	1475 (6.5)	726 (6.6)	5861 (6.5)
Small for gestational age (SGA) 2.5 percentile	793 (1.4)	293 (1.3)	145 (1,3)	1231 (1.4)
Number of children with high birth weight, *n* (%)				
High birth weight children (> 4000 g)	12,515 (21.9)	4905 (21.6)	2390 (21.8)	19,810 (21.8)
Large for gestational age (LGA) 10 percentile	6633 (11.7)	2557 (11.3)	1285 (11.8)	10,475 (11.6)
Large for gestational age (LGA) 2.5 percentile	2110 (3.7)	809 (3.6)	418 (3.8)	3337 (3.7)
Number of children with malformation, *n* (%)	2697(4.7)	1108 (4.9)	519 (4.7)	4324 (4.8)
Number of stillbirths, *n* (%)	125 (0.2)	49 (0.2)	30 (0.3)	204 (0.2)

Compared to the reference group, there was no statistically significant increased risk for any adverse birth outcomes for participants who had consulted a dentist during pregnancy without having white fillings placed or for those who had white fillings placed (Table 3).

**Table 3:** Crude and adjusted odds ratio (OR) and confidence interval (CI) for adverse birth outcomes related to dental treatment during pregnancy. (Reference category: Women who did not consult a dentist, OR = 1)

		Consulted a dentist, no white fillings placed OR (95% CI)	Consulted a dentist, white filling placed OR (95% CI)
Very preterm birth (≤ 32 weeks)			
Girls	Crude	0.96 (0.71–1.30)	0.91 (0.60–1.37)
	Adjusted	0.94 (0.69–1.27)	0.88 (0.58–1.33)
Boys	Crude	0.91 (0.69–1.21)	1.08 (0.76–1.53)
	Adjusted	0.88 (0.67–1.17)	1.02 (0.72–1.45)
All	Crude	0.92 (0.75–1.14)	1.01 (0.77–1.31)
	Adjusted	0.90 (0.73–1.11)	0.97 (0.74–1.26)
Late preterm birth (33–36 weeks)			
Girls	Crude	1.00 (0.89–1.13)	1.05 (0.90–1.22)
	Adjusted	1.00 (0.89–1.12)	1.03 (0.88–1.19)
Boys	Crude	1.06 (0.95–1.18)	1.16*(1.01–1.34)
	Adjusted	1.05 (0.94–1.18)	1.14 (0.99–1.31)
All	Crude	1.03 (0.96–1.12)	1.10 (1.00–1.23)
	Adjusted	1.03 (0.95–1.11)	1.08 (0.97–1.20)
Low birth weight (< 2500 g)			
Girls	Crude	1.01 (0.88–1.51)	1.03 (0.86–1.23)
	Adjusted	0.98 (0.86–1.12)	0.99 (0.83–1.18)
Boys	Crude	0.96 (0.84–1.11)	1.03 (0.86–1.24)
	Adjusted	0.94 (0.82–1.09)	0.99 (0.83–1.19)
All	Crude	0.99 (0.90–1.09)	1.03 (0.91–1.17)
	Adjusted	0.96 (0.87–1.06)	0.99 (0.87–1.13)
Small for gestational age (SGA) 10 percentile			
Girls	Crude	1.07 (0.97–1.17)	1.14*(1.01–1.28)
	Adjusted	1.03 (0.94–1.13)	1.10 (0.97–1.24)
Boys	Crude	0.97 (0.89–1.05)	0.95 (0.85–1.07)
	Adjusted	0.92 (0.84–1.00)	0.93 (0.83–1.04)
All	Crude	1.01 (0.95–1.08)	1.04 (0.95–1.13)
	Adjusted	0.97 (0.91–1.03)	1.00 (0.92–1.09)
Very small for gestational age (SGA) 2.5 percentile			
Girls	Crude	0.86 (0.71–1.06)	1.04 (0.81–1.34)
	Adjusted	0.84 (0.68–1.03)	0.97 (0.75–1.25)
Boys	Crude	0.98 (0.82–1.17)	0.88 (0.68–1.13)
	Adjusted	0.93 (0.77–1.11)	0.84 (0.65–1.08)
All	Crude	0.93 (0.81–1.06)	0.95 (0.80–1.14)
	Adjusted	0.89 (0.77–1.02)	0.90 (0.75–1.08)
High birth weight (> 4000 g)			
Girls	Crude	0.99 (0.94–1.05)	0.98 (0.91–1.06)
	Adjusted	1.03 (0.97–1.09)	0.98 (0.91–1.06)
Boys	Crude	0.98 (0.93–1.03)	1.01 (0.94–1.07)
	Adjusted	1.01 (0.96–1.06)	1.00 (0.93–1.07)
All	Crude	0.98 (0.94–1.02)	0.99 (0.95–1.04)
	Adjusted	1.01 (0.98–1.05)	0.99 (0.94–1.04)
Large for gestational age (LGA) 10 percentile			
Girls	Crude	0.96 (0.90–1.02)	0.99 (0.91–1.08)
	Adjusted	1.00 (0.93–1.07)	0.98 (0.90–1.08)
Boys	Crude	0.97 (0.90–1.04)	1.03 (0.94–1.13)
	Adjusted	1.01 (0.94–1.08)	1.01 (0.93–1.11)
All	Crude	0.96 (0.92–1.01)	1.01 (0.95–1.08)
	Adjusted	1.01 (0.96–1.06)	1.00 (0.94–1.06)
Large for gestational age (LGA) 2.5 percentile			
Girls	Crude	0.97 (0.87–1.09)	1.01 (0.87–1.17)
	Adjusted	1.02 (0.91–1.15)	0.99 (0.86–1.15)
Boys	Crude	0.95 (0.84–1.07)	1.06 (0.90–1.24)
	Adjusted	0.98 (0.87–1.11)	1.03 (0.88–1.20)
All	Crude	0.96 (0.88–1.04)	1.03 (0.93–1.15)
	Adjusted	1.00 (0.92–1.09)	1.01 (0.91–1.12)
Malformation			
Girls	Crude	1.02 (0.92–1.14)	0.99 (0.86–1.15)
	Adjusted	1.00 (0.90–1.12)	1.00 (0.86–1.15)
Boys	Crude	1.05 (0.95–1.15)	1.01 (0.89–1.15)
	Adjusted	1.03 (0.94–1.14)	1.00 (0.88–1.14)
All	Crude	1.03 (0.96–1.11)	1.00 (0.91–1.10)
	Adjusted	1.02 (0.95–1.09)	1.00 (0.91–1.10)
Stillbirth			
Girls	Crude	0.96 (0.59–1.55)	1.20 (0.67–2.15)
	Adjusted	0.92 (0.57–1.50)	1.16 (0.64-2.07)
Boys	Crude	0.97 (0.61–1.54)	1.30 (0.75–2.24)
	Adjusted	0.95 (0.60–1.51)	1.22 (0.70–2.11)
All	Crude	0.98 (0.71–1.37)	1.25 (0.84–1.86)
	Adjusted	0.96 (0.69–1.33)	1.18 (0.79–1.76)

The OR was adjusted for mothers age (≤19, 20–24, 25–29, 30–34, 35–39, 40+), parity (0, 1 or more previous viable pregnancies), education (≤ 12 years, 13–16 years, ≥ 17 years), pre-pregnancy body mass index (< 18.5, 18.5–24.9, 25.0–29.9, 30.0–34.9, 35.0–39.9, ≥ 40), smoking (never, occasionally, daily) and alcohol consumption during pregnancy (never, less than once a week, once a week, more than once a week). *p < 0.05

Separate analyses by gender showed that girls born to mothers who had white fillings placed during pregnancy had an increased risk of being small for gestational age (below the 10th percentile) compared to the reference group. The unadjusted OR was 1.14 (95% CI 1.01–1.28*; p =* 0.029) while after adjustment for potential confounders, the OR was reduced and not statistically significant (OR = 1.10, 95% CI 0.97–1.24; Table 3).

Boys born to mothers who received white fillings during pregnancy had a slightly increased risk of being born late preterm compared to the boys born in the reference group. The unadjusted OR was 1.16 (95% CI 1.01–1.34; *p* = 0.041), and the adjusted OR was 1.13 (95% CI 0.98–1.31; *p* = 0.082; Table 3).

## Discussion

The aim of the present study was to investigate whether the placement of polymer-based dental fillings during pregnancy was associated with outcomes including stillbirth, preterm delivery, malformations, and low or high birth weight. No evidence of an increased risk of adverse birth outcomes after placement of white fillings during pregnancy was found. Gender-specific analyses showed generally similar results for girls and boys analysed together.

The main strengths of the present study are the overall large sample size and the large number of participants who had white fillings placed. These large numbers enabled us to study even rare birth outcomes. Furthermore, the prospective design of the study reduced the risk for recall bias. Additionally, the information on health-related and lifestyle data that was derived from both the MBRN and the MoBa questionnaires enabled us to control for some potential confounding factors.

To the best of our knowledge, the present study is the first to investigate potential associations between polymer-based fillings placed during pregnancy and birth outcomes. Michalowicz et al. found no significant associations between adverse pregnancy outcomes and periodontal treatment, the use of anaesthetic during nonsurgical periodontal treatment, treatment including temporary and permanent restorations, endodontic therapy, and extractions [35]. These results are in agreement with our findings. However, in the study of Michalowicz et al., the type of restorative material was not specified. Thus, the results are not directly comparable.

A limitation of the MoBa study is the low response rate, with a possible self-selection of the healthiest women. The MoBa has an underrepresentation of young mothers (< 25 years). The participants have a higher level of education and are more likely to be non-smokers than the general population of pregnant women in Norway [36].

However, self-selection to the cohort is not a validity problem in studies of associations between exposure and outcomes [36].

The MoBa study is based on questionnaires filled in by the participating women. To achieve reliable answers from all participants in this large cohort, an effort was made to make the questions as easy and achievable as possible. Thus, information about dental treatment is sparse. Detailed information about the type and manufacturer of the polymer-based filling material and size and number of fillings placed, would be of interest. However, to obtain accurate information about this, access to dental records would be needed. In large epidemiological studies, like the MoBa study, access to updated dental records would be unfeasible. Accordingly, reliable knowledge about the number and size of possible pre-existing composite restorations is lacking. Since leakage of BPA from existing polymer-based restorative materials is very low compared with other sources [37], this information would most likely be of minor importance.

The participants were asked if they had received “white fillings” during pregnancy. In Norway, white fillings would practically be the same as polymer-based restorative fillings or so called polymer-based or resin-based composites. However, the term “white fillings” may include materials like resin-modified cements, compomers and water-based glass ionomer cements (GIC). In the period of this study, the vast majority of Norwegian dentists used polymer-based filling materials when restoring cavities in adults. Kopperud et al. described management of occlusal caries in adults by Norwegian dentists in 2009 and stated that polymer-based composite was the preferred restorative material (91.9%) [38]. In the same study the use of other filling materials was reported to be less than 4%. This is in accordance with another study examining treatment concept for approximal caries in Norway [39]. In 2009 polymer-based filling material was preferred by 94.9% of the responding dentists. Preference for other filling materials was: 1.1% compomer, 1.1% GIC, 0.5% resin-modified GIC and 1.8% a combination of resin composite and GIC [39]. In 1997, 2 years before recruitment started in MoBa, Norwegian data showed that approximately 70% of the tooth-coloured fillings placed in adults were polymer-based [40].

The participants answered questions regarding dental treatment during the first 30 weeks of pregnancy but were not asked to specify in which week of pregnancy they visited the dentist. Hence, a limitation is that we could not study if treatment with polymer-based filling materials could be a factor of importance at specific time windows during pregnancy. The severity of the effects of prenatal exposure to toxic agents appears to be influenced by the degree and timing of the exposure during gestation [41]. Some teratogens cause damage only during specific days or weeks early in pregnancy, when a particular part of the body is formed [41]. A well-known example is the thalidomide-tragedy in the late 1950s and the early 1960s, where the medication taken during days 20–36 after fertilization resulted in serious malformations of the foetus [42, 43].

Some women with the need for dental treatment do not seek or do not receive dental care during pregnancy [44]. This may, in part, be due to their concerns about the potential risk to the foetus, as well as dentists and other health care providers’ attitudes and beliefs about the safety of dental treatment during pregnancy [44].

The findings from the present study, including more than 90,000 pregnancies, are reassuring. However, taken the limitations of a prospective cohort study into account, these findings could be corroborated in case control studies. Thus, access to dental records and thereby accurate and detailed information regarding dental treatment could be possible to obtain.

## Conclusion

In this study, women who had white fillings placed during pregnancy had no increased risk for adverse birth outcomes compared with women who did not consult a dentist during pregnancy. Thus, our findings do not support the hypothesis of an association between placement of polymer-based fillings during pregnancy and adverse birth outcomes.

## Abbreviations

Bis-GMA: Bisphenol-A glycidyl dimethacrylate
BMI: Body mass index
BPA: Bisphenol A
LGA: Large for gestational age
MBRN: Medical Birth Registry of Norway
MoBa: the Norwegian Mother and Child Cohort Study
SGA: Small for gestational age
WHO: World Health Organization
